# Virus taxonomy: the database of the International Committee on Taxonomy of Viruses (ICTV)

**DOI:** 10.1093/nar/gkx932

**Published:** 2017-10-13

**Authors:** Elliot J Lefkowitz, Donald M Dempsey, Robert Curtis Hendrickson, Richard J Orton, Stuart G Siddell, Donald B Smith

**Affiliations:** Department of Microbiology, University of Alabama at Birmingham, Birmingham, AL 35294, USA; MRC-University of Glasgow Centre for Virus Research, Glasgow, UK; School of Cellular and Molecular Medicine, University of Bristol, Bristol, UK; Nuffield Department of Medicine, University of Oxford, Oxford, UK

## Abstract

The International Committee on Taxonomy of Viruses (ICTV) is charged with the task of developing, refining, and maintaining a universal virus taxonomy. This task encompasses the classification of virus species and higher-level taxa according to the genetic and biological properties of their members; naming virus taxa; maintaining a database detailing the currently approved taxonomy; and providing the database, supporting proposals, and other virus-related information from an open-access, public web site. The ICTV web site (http://ictv.global) provides access to the current taxonomy database in online and downloadable formats, and maintains a complete history of virus taxa back to the first release in 1971. The ICTV has also published the ICTV Report on Virus Taxonomy starting in 1971. This Report provides a comprehensive description of all virus taxa covering virus structure, genome structure, biology and phylogenetics. The ninth ICTV report, published in 2012, is available as an open-access online publication from the ICTV web site. The current, 10th report (http://ictv.global/report/), is being published online, and is replacing the previous hard-copy edition with a completely open access, continuously updated publication. No other database or resource exists that provides such a comprehensive, fully annotated compendium of information on virus taxa and taxonomy.

## INTRODUCTION

The process used to study any new biological entity begins by classifying it in relation to other known biological organisms, and then giving it a name (1–3). This process of classification and naming comprises the discipline of taxonomy, and has been an integral part of biological research since at least the 18th Century when Carl Linnaeus defined the principles of modern taxonomy (4). Taxonomic classification is a scientific endeavor whereby biological organisms are grouped together and placed into their proper taxonomic hierarchy based on the characteristics that form a unique descriptor identifying a particular organism. This research process is driven by individual scientists who publish their work, providing their evidence for the proposed classification. As new data are obtained either on the organisms previously studied, or on related organisms, the classification hierarchy may change. The principles, procedures, and nomenclature used to name taxa, is handled by one of the international organizations charged with developing the necessary guidelines. For example, naming of animal species is subject to the principles established by the International Commission on Zoological Nomenclature (2,5). Naming of bacterial species is guided by the International Committee on Systematic Bacteriology (3).

The taxonomic classification of viruses and naming of virus taxa is the responsibility of the International Committee on Taxonomy of Viruses (ICTV) (6). The ICTV is charged with the task of developing, refining, and maintaining a universal virus taxonomy by the Virology Division of the International Union of Microbiological Societies (7). Unlike for other groups of biological organisms, the ICTV charge extends not only to developing the guidelines for naming of taxa, but to establishing guidelines for taxonomic classification of viruses, and approving the proposed taxonomy and names before they become official. The rules governing the ICTV and its operations are defined by the ICTV Statutes (http://ictv.global/statutes.asp), while the rules for creating and naming virus taxa are provided in the International Code of Virus Classification and Nomenclature (http://ictv.global/codeOfVirusClassification.asp).

The ICTV was established in 1966 as the International Committee on Nomenclature of Viruses, and was renamed the International Committee on Taxonomy of Viruses in 1977 (6,8,9). The ICTV consists of a series of officers, subcommittee chairs, and elected members that oversee taxonomic classification and naming. There are six subcommittees, each of which covers a different group of viruses, differentiated by the type of host the virus infects (animal, plant, fungal and protist, or bacterial and archaeal) and the molecular composition of the virus genome (DNA, RNA, double or single stranded and translational polarity). Each subcommittee contains a series of study groups, generally one per virus family, that develop the demarcation criteria used to define each new taxon, and evaluate proposals using those criteria for classification and naming. These demarcation criteria are based upon the genetic and biological properties of the taxon members, and cover each of the taxonomic ranks currently recognized by the ICTV: order, family, subfamily, genus and species.

In addition to overseeing taxonomic decisions, the ICTV is also charged with disseminating these decisions to the scientific community. Therefore, the ICTV maintains a database that stores the virus taxonomy, taxon names, and associated metadata, including information on exemplar viruses for each named species. The ICTV web site (http://ictv.global) provides access to its taxonomy database and also provides an extensive collection of information supporting its overall activities, including the ICTV Report that provides a complete description of every defined virus taxon.

## DATABASE AND WEB SITE

### Virus classification

Classification of viruses is based on the collection and comparison of various characters that describe the virus, and can then be used to distinguish one virus from another (1,10–15). Characters can consist of any property or feature of the virus, and include the molecular composition of the genome; the structure of the virus capsid and whether or not it is enveloped; the gene expression program used to produce virus proteins; host range; pathogenicity; and sequence similarity. While all characters are important in determining taxonomic relationships, sequence comparisons using both pairwise sequence similarity and phylogenetic relationships have become one of the primary sets of characters used to define and distinguish virus taxa.

Based on an assessment of characters, a hierarchical relationship is established that groups together viruses with similar properties. The properties that define higher-level taxa are shared with all lower-level taxa that belong to higher-level taxa (15). For example, viruses of the order *Picornavirales* all are non-enveloped, icosahedral particles containing one or two segments of positive-sense RNA (16). The family *Picornaviridae* is one of (currently) five families belonging to the order *Picornavirales*. Members of the family *Picornaviridae* contain a single monocistronic genome with conserved genome organization (arrangement of encoded polypeptides). Members of the genus *Enterovirus* (family *Picornaviridae*) share more than 42% amino acid identity across the length of their polyprotein. Finally, members of the species *Enterovirus C*, share a limited range of hosts and host receptors, have similar polyprotein processing programs, and share more than 70% amino acid identity in the polyprotein. Once these criteria for grouping similar viruses into common taxa are established, they are used as demarcation criteria to determine if newly discovered viruses are members of an existing species (and therefore also in existing higher-level taxa), or if the new viruses warrant creation of a new species (and potentially new higher-level taxa), if their properties are sufficiently distinct from the viruses in an existing species.

### The ICTV database of virus taxonomy

Every year, virologists submit proposals to the ICTV recommending the creation of new taxa as well as renaming, moving, or abolishing existing taxa. These proposals are submitted to the appropriate study group which then evaluates and modifies the proposal as necessary, in consultation with the original author. Once a year, the ICTV Executive Committee (EC) meets, discusses all submitted proposals, and will either accept a proposal, send it back for modification, or reject it. Then the set of accepted proposals are sent to the ICTV membership for ratification. Once ratified, the newly approved taxonomy is entered in the ICTV taxonomic database. The database utilizes a relational schema built using Microsoft SQL Server. The tables store a controlled vocabulary used for specific fields where the possible data values remain fairly constant. These include taxonomy level (currently species, genus, subfamily, family, and order), possible taxonomic changes from the previous release (new, split, move, merge, rename, and abolish), and virus genome type (e.g. dsDNA, ssDNA, dsRNA, ssRNA(+), ssRNA(–), etc.) that forms part of the annotation available for the member viruses of each species. Each record in the taxonomy table stores data on a single taxon including its name, numerical id, parent taxon, release number and date, most recent taxonomic change, filename of the proposal establishing the taxon, and a pointer to its previous record if a change has occurred in taxon data (such as moving a species to another genus). With this information, a complete history of every taxon can be reconstructed from the database. Table 1 displays an example set of database records for the species *Measles morbillivirus*.

**Table 1.** Database Records for the Species *Measles morbillivirus*

Table Field	Record 1	Record 2
taxon_name	Measles virus	Measles morbillivirus
taxon_level	Species	Species
release_number	30	31
release_year	2015	2016
taxon_id (stable)	19750163	19750163
node_id (new with each release)	20151044	20161044
parent_taxon	Morbillivirus	Morbillivirus
last_change	move	rename
proposal	2015.011a-iM.A.v2.Pneumoviridae.pdf	2016.011aM.A.v2.Paramyxoviridae_spren.pdf

The two most recent records in the ICTV database are displayed, detailing changes in the taxonomic classification and naming of virus members of this species.

**Figure 1.**
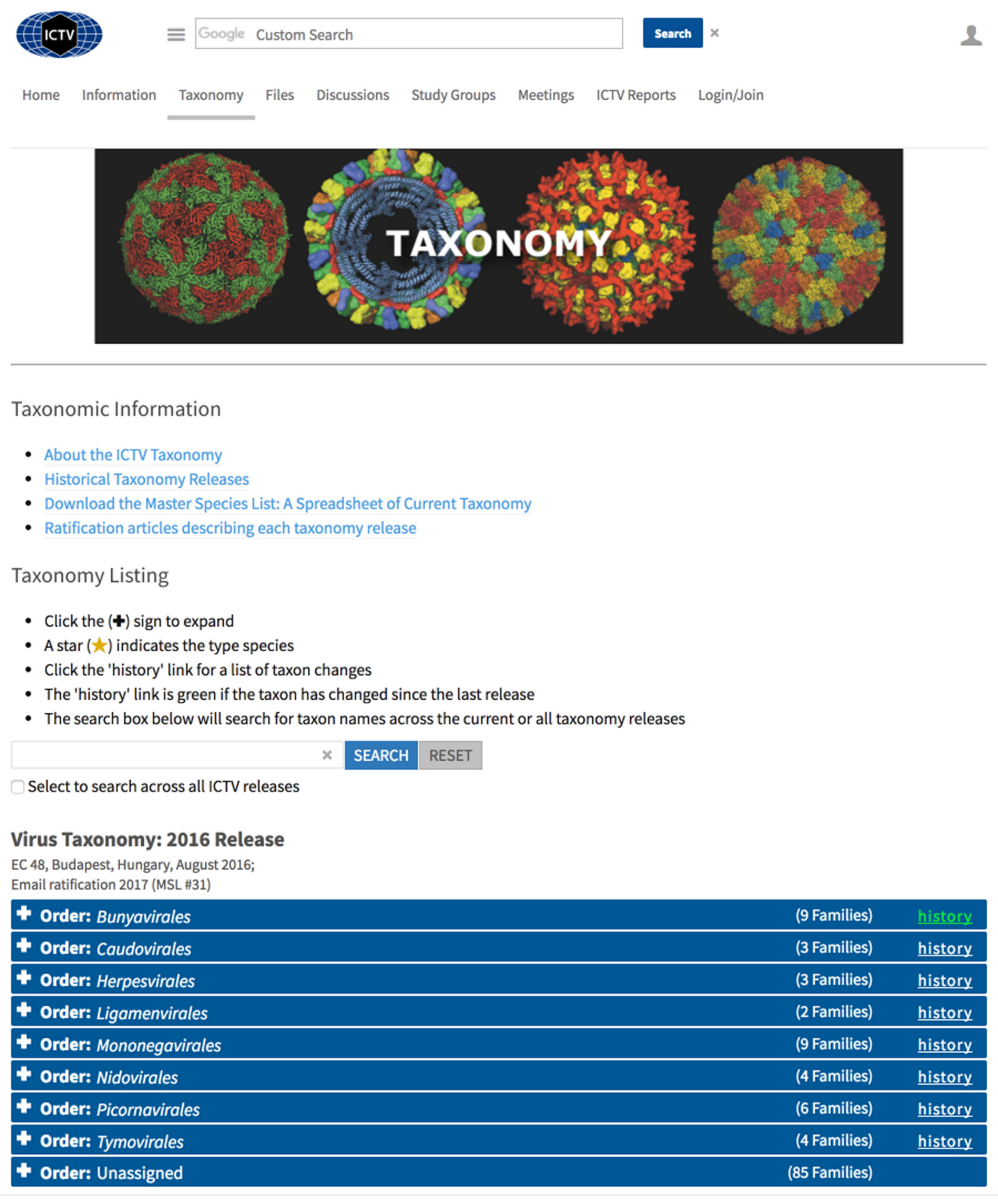
Taxonomy Web Page. A screen shot of the ICTV web page (http://ictv.global/virusTaxonomy.asp) that contains the taxonomy browser and search form.

**Figure 2.**
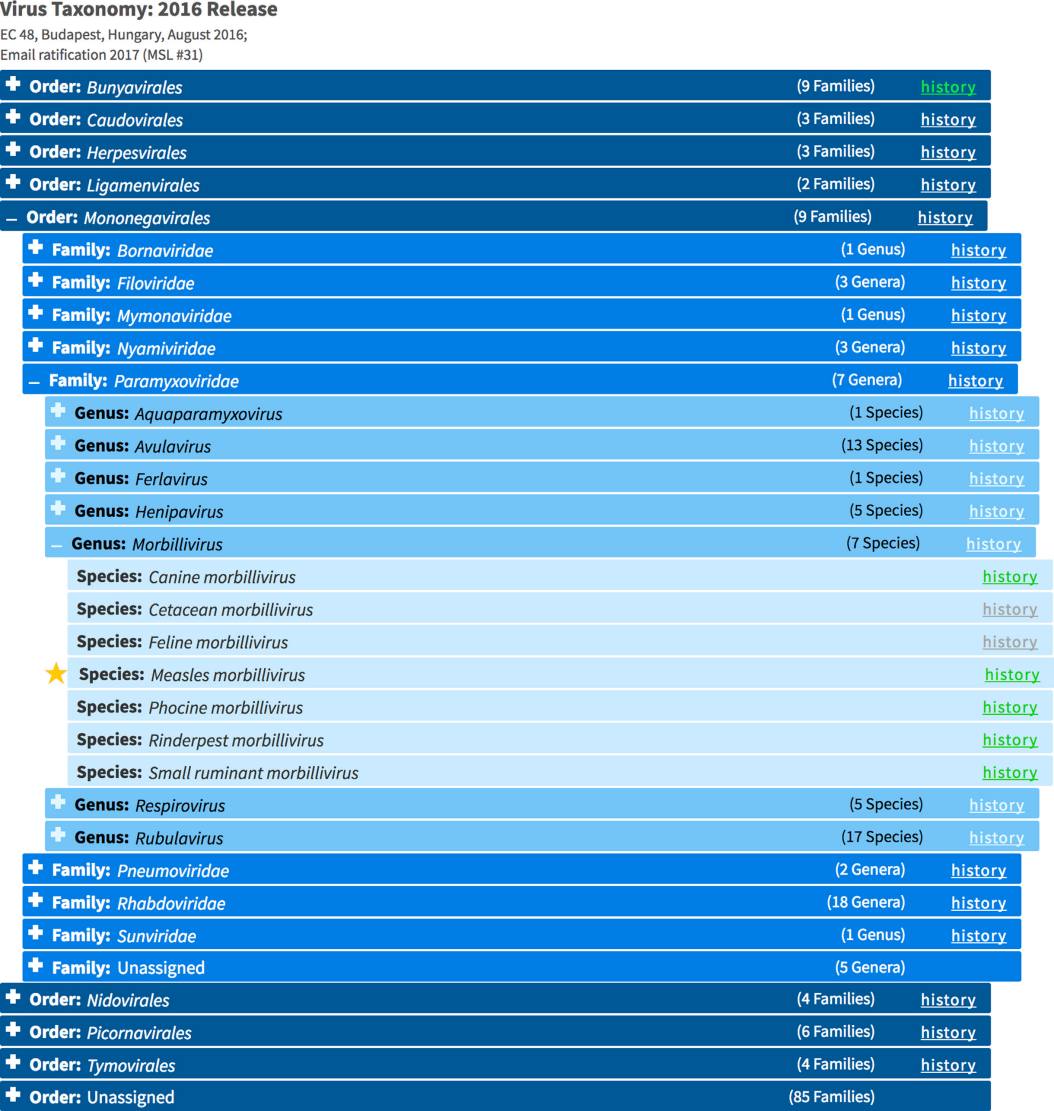
Taxonomy browser. The online taxonomy browser, showing an expanded view of the taxonomic hierarchy of the species *Measles morbillivirus*. The ‘history’ links to the right of each row are colored green if there has been a change in the indicated taxon since the last release. Clicking on any ‘history’ link will take the user to the taxon history page (Figure 3).

Data stored in the taxonomy database can be accessed visually from the ICTV web site (http://ictv.global/virusTaxonomy.asp) using the taxonomy browser: a hierarchical series of expandable, tabular rows, with each row containing information on a single taxon (Figure 1). Each taxon is presented in its proper position in the taxonomic hierarchy (Figure 2). Each row is labeled with the number of lower-level taxa belonging to each higher-level taxon, and a link is provided to the complete history of each taxon (see below). A search box is available to search the current, or current and historical taxonomy releases for any taxon that includes the text entered in the search box (Supplementary Figure S1). Each search result is linked to the identified taxon in the hierarchical taxonomy browser.

The complete taxonomy for any one release (the Master Species List) is also available for download as an Excel spreadsheet (http://ictv.global/msl.asp). These spreadsheets are available for every release back to 2008. Each new taxonomy release is also shared with the National Center for Biotechnology Information (NCBI) of the National Institutes of Health so that the taxonomy database they maintain and use for the annotation of virus sequence records can be kept up-to-date with the most recent ICTV taxonomy.

### Historical taxonomy releases

**Figure 3.**
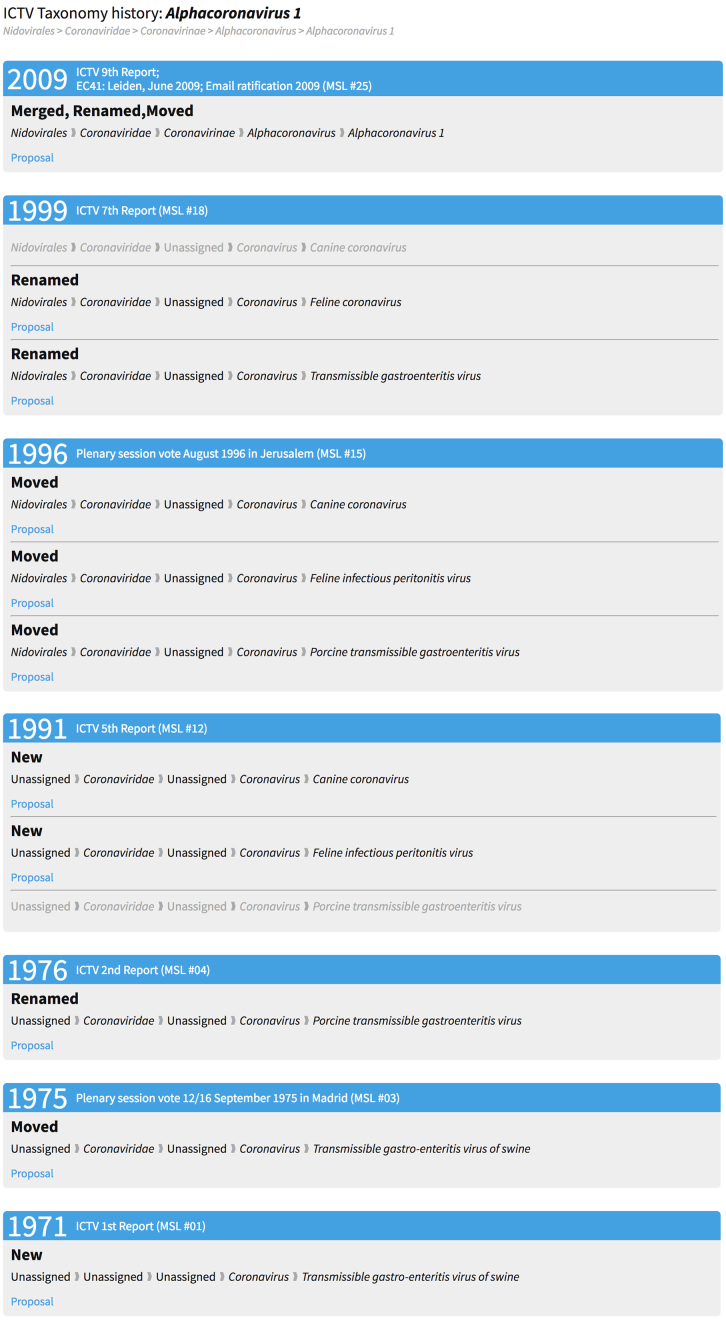
Taxon history. Complete history of the species *Alphacoronavirus 1*. The bottom of the figure displays the earliest taxon (transmissible gastro-enteritis virus of swine) into which viruses belonging to the current taxon, *Alphacoronavirus 1*, were classified. Moving up the page provides an account of every change that has occurred including moves to new higher-level taxa, new names, and merges with other existing species. Within a particular release, a grayed-out row indicates that there was no change to that taxon, while other rows depict changes as indicated by the label above the row (New, Moved, Renamed and Merged).

The ICTV database contains the complete taxonomy for every release since the taxonomy was first produced in 1971 (Table 2) (6,8). Each of these releases can be accessed using the taxonomy browser by following the appropriate link on the release history web page (http://ictv.global/taxonomyReleases.asp). In addition, by selecting the ‘history’ link from the taxonomy browser (Figure 2), a page displaying the complete history of the selected taxon is displayed (Figure 3). This history is constructed from the complete set of database records that are part of the historical path leading to the selected taxon, and to the most recent child of that taxon in the last release recording a change. For example, in Figure 3, the most recent change to this particular taxonomic lineage was the creation of the species *Alphacoronavirus 1* in 2009. But in fact, this was not a new species, but was a merging of three existing species (transmissible gastroenteritis virus, feline coronavirus, and canine coronavirus) into one, newly renamed species, *Alphacoronavirus 1*. In addition, the species were moved from the now defunct genus coronavirus, into the genus *Alphacoronavirus* that is a member of the subfamily *Coronavirinae*. This particular history goes back to the first ICTV data release in 1971 when the species transmissible gastro-enteritis virus of swine was created as a member of the genus coronavirus. (The species concept in virus taxonomy was not defined until 1996 (8). Therefore any ‘species’ present in the database between 1971 and 1995 represents an informal taxonomic rank, existing to contain the viruses that are members of each genus and higher-level ranks.)

**Table 2.** Taxonomy Release History. Information on each release of the approved ICTV taxonomy is displayed including the date and location of the ICTV meeting where proposals were discussed; the Master Species List (MSL) release number and the number of taxa belonging to each taxon rank present in the ICTV database for the indicated release

		Number of Taxa				
Year	Release Information	Order	Family	Subfamily	Genus	Species
2016	EC 48, Budapest, Hungary, August 2016; Email ratification 2017 (MSL #31)	8	122	35	735	4404
2015	EC 47, London, UK, July 2015; Email ratification 2016 (MSL #30)	7	111	27	609	3704
2014	EC 46, Kingston and Montreal, Canada, July 2014, Email ratification 2015 (MSL #29)	7	104	23	505	3185
2013	EC 45, Edinburgh, July 2013; Email ratification 2014 (MSL #28)	7	103	22	455	2827
2012	EC 44, Leuven, July 2012; Email ratification 2013 (MSL #27)	7	96	22	420	2617
2011	EC 42: Paris, June 2010; EC43: Sapporo, September 2011; Email ratification 2012 (MSL #26)	6	94	22	395	2480
2009	ICTV 9th Report; EC41: Leiden, June 2009; Email ratification 2009 (MSL #25)	6	87	19	349	2285
2008	EC 39: Kingston, June 2007; EC 40: Istanbul, August 2008; Email ratification 2008 (MSL #24)	5	82	11	307	2079
2005	ICTV 8th Report (MSL #23)	3	73	11	289	1899
2004	Postal vote 2004 (MSL #22)	3	73	11	290	1832
2002	Plenary session, July 2002, Paris (MSL #21)	3	70	11	251	1619
2002	Postal vote spring 2002 (MSL #20)	3	70	9	247	1602
1999	Plenary session, August 1999, Sydney (MSL #19)	3	64	9	239	1550
1999	ICTV 7th Report (MSL #18)	3	64	9	234	1551
1998	Postal vote autumn 1998 (MSL #17)	3	63	9	233	2370
1997	Postal vote autumn 1997 (MSL #16)	2	56	9	197	2267
1996	Plenary session, August 1996, Jerusalem (MSL #15)	2	53	9	182	2253
1995	ICTV 6th Report (MSL #14)	1	50	9	166	2220
1993	Plenary session, August 1993, Glasgow (MSL #13)	1	49	9	160	1700
1991	ICTV 5th Report (MSL #12)	1	40	9	142	1674
1990	Plenary session, August 1990, Berlin (MSL #11)	1	40	9	137	1290
1987	Plenary session, August 1987, Edmonton (MSL #10)	0	37	8	116	1275
1984	Plenary session, September 1984, Sendai (MSL #09)	0	35	8	103	1222
1982	ICTV 4th Report (MSL #08)	0	29	8	97	1209
1981	Plenary session, August 1981, Strasbourg (MSL #07)	0	29	8	93	1091
1979	ICTV 3rd Report (MSL #06)	0	24	8	84	1008
1978	Plenary session, August 1978, The Hague (MSL #05)	0	24	7	76	760
1976	ICTV 2nd Report (MSL #04)	0	17	3	67	754
1975	Plenary session, September 1975, Madrid (MSL #03)	0	17	1	63	309
1974	Postal vote April-May 1974 (MSL #02)	0	5	0	49	298
1971	ICTV 1st Report (MSL #01)	0	2	0	43	290

Each change indicated in the historical taxonomic record is documented by the taxonomy proposal that was submitted to the ICTV proposing that change. These proposals are accessible from the ICTV web site, and can be downloaded as pdf files (Supplementary Figure S2). Links to each relevant proposal are provided from the taxonomy history web page (Figure 3). Proposals are available for all taxonomic changes since 2008; though many earlier proposals are also available.

### ICTV Online (10th) Report on virus taxonomy

In addition to providing current and historical access to the ICTV taxonomy through the web-based browser and the Master Species List, the ICTV has also published a compendium, the ‘ICTV Reports on Virus Taxonomy: The Classification and Nomenclature of Viruses’ since 1971, providing a description of all virus taxa existing at the time of publication (1,9,17,18). This has been published as a hard-copy book, and contains a complete listing of all taxa, along with a list of exemplar viruses corresponding to each species. In addition, these reports provide extensive information on each taxon, describing the properties and characteristics of the virus members of each taxon. These reports also contain a description of the demarcation criteria used to determine whether a particular virus should be classified into an existing species, or whether the creation of a new species (and potentially higher-level taxa) is warranted. The last hard-copy ICTV Report published was the ninth Report, published in 2012 (1). (Due to the length of time required to compile the report, the ninth Report was based on the 2009 ICTV taxonomy release.) The ninth report is currently available as an open-source publication from the ICTV web site (http://ictv.global/9th-report/). The ICTV 10th Report on Virus Taxonomy is being published in an online format, and is available from the ICTV web site (http://ictv.global/report/). The 10th report is being published sequentially as chapters are released at the rate of ∼25 a year.

**Figure 4.**
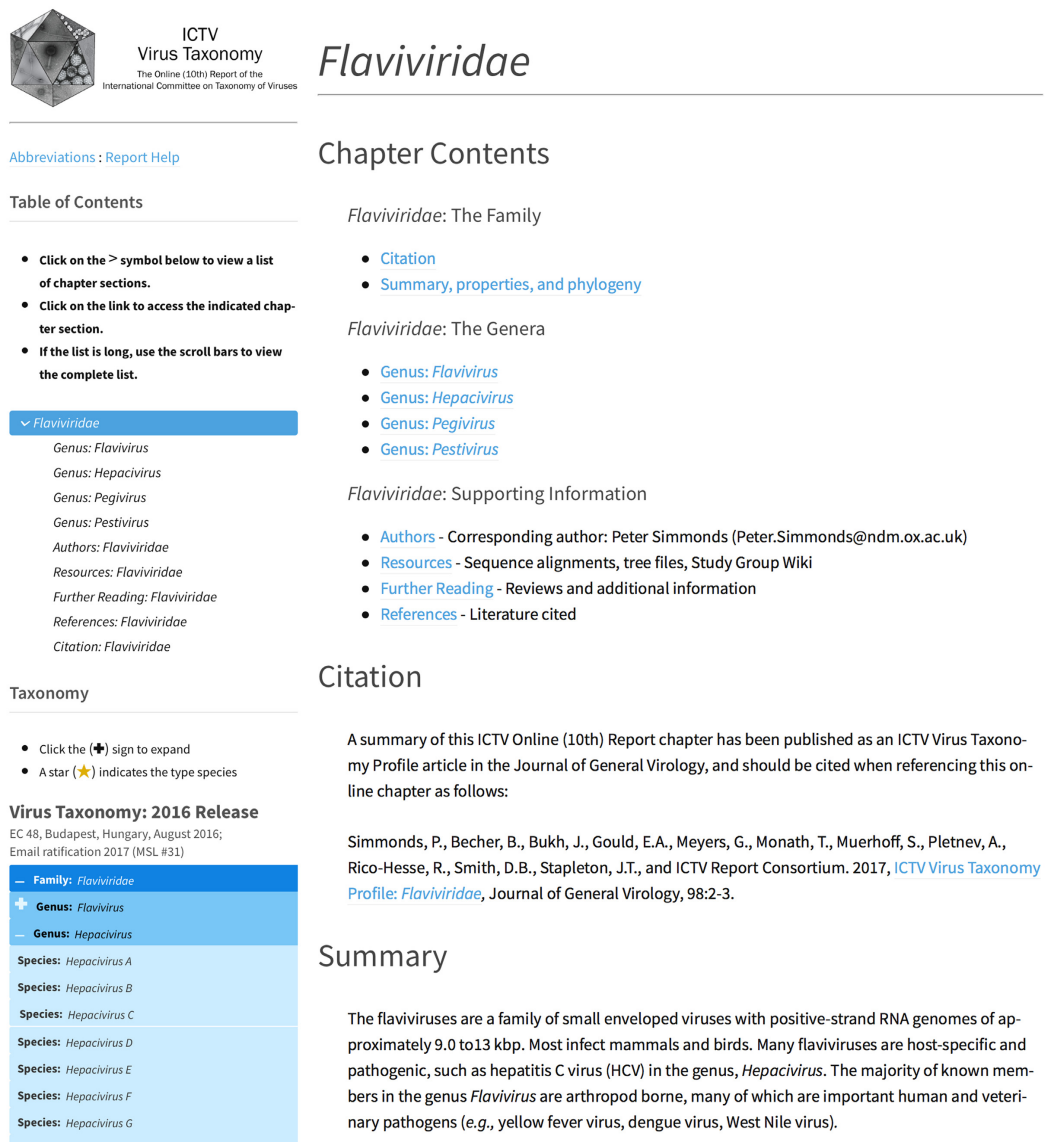
ICTV Report chapter. A screen shot of the web page depicting the *Flaviviridae* chapter of the ICTV Online (10th) Report (http://ictv.global/report/flaviviridae/).

Report chapters provide a thorough description of each virus taxon, and are generally organized by taxonomic family (Figure 4). Within each family, separate sections describe each genus and the member species of that genus. Each of these sections contains a member species table that provides a list of species, and one exemplar virus corresponding to that species. These exemplars are not meant to be a comprehensive listing of all member viruses of the species, but comprise viruses that can serve as a prototypic member of each species. These tables are populated from information stored within the ICTV database, and may also provide names of additional prototypic virus isolates, as well as accession number-based links to available GenBank (19) sequence records (Supplementary Table S1). Accession numbers for RefSeq (20) sequence records are also stored in the database and will be made available in a future update of the web site.

In addition to the Online Report, the ICTV is also partnering with the Journal of General Virology to publish short summaries of each report chapter (21). These ICTV Virus Taxonomy Profiles are published at the same time as the Online Report chapters, and consist of a two to three-page review of the properties of each virus family. These profiles serve as citable references for the Online Report chapters (22).

## FUTURE DEVELOPMENTS

A number of challenges currently exist for the ICTV and virus taxonomy that generally derive from our nascent understanding of the true extent of worldwide virus diversity, arising from new and more efficient technologies for discovering, measuring, and characterizing that diversity (10,23–29). Virus discovery has been one of the areas of emphasis of large environmental sampling and metagenomic sequencing projects. The goal of these projects is to more thoroughly assess, in an unbiased manner, the true diversity of biological organisms in a particular environment at a particular place in time, by sequencing the genomes of all organisms present in a sample using high-throughput sequencing technologies. For viruses, this entails utilizing methods that sequence not only DNA-based organisms, but organisms (viruses) with RNA genomes, by including a reverse transcription step in the metagenomic isolation and sequencing pipeline. Once the sequence reads have been generated, efficient sequence assemblers are then able to then identify the reads unique to a single virus genome, and assemble them into a complete, or almost complete, genomic sequence. These metagenomic sequencing projects have resulted in the assembly of thousands of new virus genomes that when classified, should result in the creation of thousands of new virus species and higher-level taxa.

In June of 2016, the ICTV helped organize a meeting to discuss metagenomic sequencing and its impact on virus classification. A consensus statement published by meeting participants recommended that viruses detected solely by metagenomic sequencing contain sufficient information in their genomic sequence to support their classification and naming (30,31). The ICTV concurred with that recommendation. Therefore, the ICTV is now accepting proposals for new virus taxa supported solely by metagenomic sequence information. This policy should ensure that the official ICTV virus taxonomy will be much more reflective of the true diversity of existing viruses. But at the same time, this will require that the systems in place for characterizing virus diversity, proposing new virus taxa, storing these taxa in the ICTV database, and browsing and searching for these taxa from the ICTV web pages, be streamlined and made capable of handling the many thousands of new taxa that will need to be created. The ICTV may also need to modify its policies and procedures for naming of virus species and expand the available taxonomic ranks needed to classify these viruses, to handle the large number of new, diverse viruses discovered using metagenomic sequencing methods.

In the past, the ICTV has continually modified and modernized its approach to virus taxonomy through, for example, moving to sequence-based characters as the primary source of information needed for classification (30); developing and utilizing automated computational methods to facilitate the grouping of viruses into taxa (existing and new) based on a comparison of their genomic sequences (10,32–35); operating more efficiently and shortening the time taken to approve proposals for new taxa; and now, basing taxonomic decisions solely on the information contained in genomic sequences (30). It is only through this process of continued evaluation of the current state of virus discovery and research, responding to new data and new understanding by updating policies and procedures, that the ICTV will be able to remain current and continue to be relevant to the scientific community.

## AVAILABILITY

The ICTV web site and all applications accessing the ICTV database are available from http://ictv.global/. The database and web site are the responsibility of the ICTV Data Secretary, an elected position of the ICTV membership serving a 6-year term. This position, and the provision of the database and web site are defined within and required by the ICTV Statutes (http://ictv.global/statutes.asp), thus assuring the long-term existence of this resource. Unless otherwise noted, all information provided by the ICTV from its web site are provided under the Creative Commons Attribution-ShareAlike 4.0 International license (https://creativecommons.org/licenses/by-sa/4.0/).

## Supplementary Material

Supplementary Data
