# Supplementary material for: Virus taxonomy: the database of the International Committee on Taxonomy of Viruses (ICTV)
Source: Nucleic Acids Res. 2017 Oct 13;46(Database issue):D708–17. doi: 10.1093/nar/gkx932 (PMC5753373; doi:10.1093/nar/gkx932)
Supplement: Supplementary Data [file gkx932_supp.pdf]

SUPPLEMENTARY TABLE AND FIGURE LEGENDS

Supplementary Table 1. *Hepacivirus* Member Species

The member species table, provided within the ICTV Online (10th) Report chapters, provides the indicated information on exemplar viruses for each species. The species *Hepacivirus C*, also contains information on additional member viruses that have been classified by the hepacivirus research community as prototypic members of important clades of viruses that may have unique pathogenic, epidemiological, or other biological properties (1, 2).

1. Smith, D.B., Bukh, J., Kuiken, C., Muerhoff, A.S., Rice, C.M., Stapleton, J.T. and Simmonds, P. (2014) Expanded classification of hepatitis C virus into 7 genotypes and 67 subtypes: updated criteria and genotype assignment web resource. Hepatology, 59, 318-327.

2. Simmonds, P., Bukh, J., Combet, C., Deleage, G., Enomoto, N., Feinstone, S., Halfon, P., Inchauspe, G., Kuiken, C., Maertens, G. et al. (2005) Consensus proposals for a unified system of nomenclature of hepatitis C virus genotypes. Hepatology, 42, 962-973.

Supplementary Figure 1. Taxon search. Results from the taxonomy search interface provided from the taxonomy browser web page (http://ictv.global/virusTaxonomy.asp). The search set can comprise the entire history of ICTV taxonomy releases, or just the current release. The ‘View’ link will take the user to the row of the indicated release year in the taxonomy browser for the indicated taxon.

Supplementary Figure 2. Taxonomic proposals. This page provides a list of approved proposals available for download from the ICTV web site file repository (http://ictv.global/Official_ICTV_Proposals.asp) that detail the justification for each taxonomic change.

Supplementary Table 1. *Hepacivirus* Member Species

Species	Virus name(s)	Exemplar isolate	Exemplar accession number	Available sequence*	Other isolates	Other isolate accession numbers	Virus Abbreviation(s)
Hepacivirus A	Non-primate hepacivirus	NZP1	KP325401	CG			NZP1
Hepacivirus B	GB virus-B	GBV-B-T1053	U22304	CG			GBV-B
Hepacivirus C	hepatitis C virus genotype 1a	H77	AF011751	CG			HCV-H77
	hepatitis C virus genotype 1a				HCV-PT	M62321	HCV-PT
	hepatitis C virus genotype 1b				J	D90208	HCV-J
	hepatitis C virus genotype 2a				HC-J6	D00944	HCV-HC-J6
	hepatitis C virus genotype 2b				HC-J8	D01221	HCV-HC-J8
	hepatitis C virus genotype 3a				NZL-1	D17763	HCV-NZL-1
	hepatitis C virus genotype 3k				JK049	D63821	HCV-JK049
	hepatitis C virus genotype 4a				ED43	GU814265	HCV-ED43
	hepatitis C virus genotype 5a				EUH1480	Y13184	HCV-EUH1480
	hepatitis C virus genotype 6a				EUHK2	Y12083	HCV-EUHK2

	hepatitis C virus genotype 6g				JK046	D63822	HCV-JK046
	hepatitis C virus genotype 7a				QC-69	EF108306	HCV-QC69
Hepacivirus D	Guereza hepacivirus	BWC08	KC551800	CCG			GHV-1-BWC08
Hepacivirus E	rodent hepacivirus	339	KC815310	CG			RHV-339
Hepacivirus F	rodent hepacivirus	NLR07-oct70	KC411784	CCG			NLR07-oct70
Hepacivirus G	Norway rat hepacivirus 1	NYC-C12	KJ950938	CG			NYC-C12
Hepacivirus H	Norway rat hepacivirus 2	NYC-E43	KJ950939	CG			NYC-E43
Hepacivirus I	rodent hepacivirus	SAR-3/RSA/2008	KC411806	CCG			SAR-3-RSA-2008
Hepacivirus J	rodent hepacivirus	RMU10-3382/GER/2010	KC411777	CCG			RMU10-3382-GER-2010
Hepacivirus K	bat hepacivirus	PDB-829	KC796074	CCG			PDB 829
Hepacivirus L	bat hepacivirus	PDB-112	KC796077	CCG			PDB-112
Hepacivirus M	bat hepacivirus	PDB-491.1	KC796078	CCG			PDB491.1
Hepacivirus N	bovine hepacivirus	463/Ger/2014	KP641127	CCG			463-GER-2014

*CG: Complete Genome; CCG: Coding Complete Genome

Supplementary Figure 1. Taxon search.

Taxonomy Listing

- Click the (✚) sign to expand
- A star (★) indicates the type species
- Click the 'history' link for a list of taxon changes
- The 'history' link is green if the taxon has changed since the last release
- The search box below will search for taxon names across the current or all taxonomy releases

hepatitis ✕ SEARCH RESET

☐ Select to search across all ICTV releases

	Release	Level	
VIEW	2016	Species	Unassigned->*Hepadnaviridae*->*Avihepadnavirus*->*Duck hepatitis B virus*
VIEW	2016	Species	Unassigned->*Hepadnaviridae*->*Avihepadnavirus*->*Heron hepatitis B virus*
VIEW	2016	Species	Unassigned->*Hepadnaviridae*->*Avihepadnavirus*->*Parrot hepatitis B virus*
VIEW	2016	Species	Unassigned->*Hepadnaviridae*->*Orthohepadnavirus*->*Ground squirrel hepatitis virus*
VIEW	2016	Species	Unassigned->*Hepadnaviridae*->*Orthohepadnavirus*->*Hepatitis B virus*
VIEW	2016	Species	Unassigned->*Hepadnaviridae*->*Orthohepadnavirus*->*Long-fingered bat hepatitis B virus*
VIEW	2016	Species	Unassigned->*Hepadnaviridae*->*Orthohepadnavirus*->*Pomona bat hepatitis B virus*
VIEW	2016	Species	Unassigned->*Hepadnaviridae*->*Orthohepadnavirus*->*Roundleaf bat hepatitis B virus*
VIEW	2016	Species	Unassigned->*Hepadnaviridae*->*Orthohepadnavirus*->*Tent-making bat hepatitis B virus*
VIEW	2016	Species	Unassigned->*Hepadnaviridae*->*Orthohepadnavirus*->*Woodchuck hepatitis virus*
VIEW	2016	Species	Unassigned->*Hepadnaviridae*->*Orthohepadnavirus*->*Woolly monkey hepatitis B virus*
VIEW	2016	Species	Unassigned->*Hepadnaviridae*->Unassigned->*White sucker hepatitis B virus*
VIEW	2016	Species	Unassigned->Unassigned->*Deltavirus*->*Hepatitis delta virus*

Supplementary Figure 2. Taxonomic proposals.

Animal DNA Viruses and Retroviruses

Options

View all

View slideshow

By date | Descending

2016.021aD.A.v1.Lentivirus_sp.pdf
40 downloads View
amqking Mar 19, 2017

2016.020aD.A.v1.Gammaretrovirus_sp.pdf
28 downloads View
amqking Mar 19, 2017

2016.019aD.A.v1.Orthohepadnavirus_sp.pdf
39 downloads View
amqking Mar 19, 2017

2016.018a-dD.A.v3.Centapoxvirus.pdf
27 downloads View
amqking Mar 19, 2017

2016.017aD.A.v2.Hepadnaviridae_sp.pdf
23 downloads View
amqking Mar 19, 2017

2016.016aD.A.v1.Betapolyomavirus_2sp.pdf
25 downloads View
amqking Mar 19, 2017

2016.015aD.A.v1.Betapolyomavirus_sp.pdf
24 downloads View
amqking Mar 19, 2017

2016.014aD.A.v1.Alphapolyomavirus
amqking Mar 19, 2017
